# PKMζ Differentially Utilized between Sexes for Remote Long-Term Spatial Memory 

**DOI:** 10.1371/journal.pone.0081121

**Published:** 2013-11-11

**Authors:** Veronica Sebastian, Tatyana Vergel, Raheela Baig, Lisa M. Schrott, Peter A. Serrano

**Affiliations:** 1 Department of Psychology, Hunter College, New York, New York, United States of America; 2 Department of Pharmacology, Toxicology and Neuroscience, Louisiana State University Health Sciences Center, Shreveport, Louisiana, United States of America; 3 The Graduate Center of CUNY, New York, New York, United States of America; SUNY Downstate Medical Center, United States of America

## Abstract

It is well established that male rats have an advantage in acquiring place-learning strategies, allowing them to learn spatial tasks more readily than female rats. However many of these differences have been examined solely during acquisition or in 24h memory retention. Here, we investigated whether sex differences exist in remote long-term memory, lasting 30d after training, and whether there are differences in the expression pattern of molecular markers associated with long-term memory maintenance. Specifically, we analyzed the expression of protein kinase M zeta (PKMζ) and the α-amino-3-hydroxy-5-methyl-4-isoxazolepropionic acid (AMPA) receptor subunit GluA2. To adequately evaluate memory retention, we used a robust training protocol to attenuate sex differences in acquisition and found differential effects in memory retention 1d and 30d after training. Female cohorts tested for memory retention 1d after 60 training trials outperformed males by making significantly fewer reference memory errors at test. In contrast, male cohorts tested 30d after 60 training trials outperformed females of the same condition, making fewer reference memory errors and achieving significantly higher retention test scores. Furthermore, given 60 training trials, females tested 30d later showed significantly worse memory compared to females tested 1d later, while males tested 30d later did not differ from males tested 1d later. Together these data suggest that with robust training males do no retain spatial information as well as females do 24h post-training but maintain this spatial information for longer. Males also showed a significant increase in synaptic PKMζ expression and a positive correlation with retention test scores, while females did not. Interestingly, both sexes showed a positive correlation between retention test scores and synaptic GluA2 expression. Furthermore, the increased expression of synaptic PKMζ, associated with male memory but not with female memory, identifies another potential sex-mediated difference in memory processing.

## Introduction

Sex differences in learning and memory function have been identified across a number of species and paradigms [1,2]. Because learning and memory is such an important and basic survival function, it is intriguing that this effect is so prevalent. Since the first published reports on sex differences in learning and memory, substantial progress has been made in delineating both their behavioral effects and neurochemical underpinnings. It has been postulated that these differences evolved as a consequence of the mating practices of animals, whereby polygamous species require larger spatial navigational skills compared to monogamous ones [3,4]. Specifically, monogamous species demonstrate similar territory size for male and female pairs, thereby requiring similar predispositions for spatial navigation and memory. In contrast, in polygynous species, males have larger territories than females, a predisposition identified in meadow voles, rats and humans. Recently, this interpretation has been challenged by a proposal that sex differences in spatial ability are merely side effects of testosterone, comparable to male pattern baldness and acne [2].

Regardless of the evolutionary origins of this phenomenon, the consequences on learning and memory have been robustly documented. Many studies report male rodents as having a stronger aptitude for spatial information and a strong preference for hippocampal-based place strategy compared to a striatal-based procedural strategy [5–8]. Additional studies have identified that females use fewer hippocampal-dependent strategies [9–11], but greater levels of overall activation during spatial training compared to males [12]. Since female rodents do not prefer a hippocampal-based strategy, spatial tasks may be more difficult to learn [13] and may also down-regulate neurogenesis [14]. In the case of water maze acquisition, males outperformed females during acquisition but that advantage did not translate to improved retention during the probe trials [10,15]. Furthermore, the male learning strategy is not susceptible to hormone manipulations early in development, which affects accuracy during training, but not strategy preference [16]. In contrast, females are influenced by fluctuating gonadal hormones, which improve learning during the proestrus stage of their cycle [17,18]. These studies also demonstrate a male advantage during the learning / acquisition phase across a few days. The short training periods can exacerbate the effects of hormones, depending on when in the estrus cycle the training was given. To accommodate for these hormone effects, we chose to investigate sex effects on remote (30d) long-term memory that requires 6 days of training. The long training period ensures that females in every cohort experience training across all parts of the cycle, minimizing the effects of fluctuating hormones. 

We utilized a spatial paradigm on the radial arm maze (RAM) using two training protocols, involving 30 or 60 trials that minimize sex differences in acquisition. This allowed us to examine sex differences in memory retrieval, without the confounding effects of sex differences in acquisition. We looked at two retention time points, 1d or 30d after completing training, to examine how sex differences impact long-term retrieval. The radial arm maze was chosen for two primary reasons: (1) to avoid the inherently stressful nature of the water maze [14,19] and (2) to test two memory systems, reference and working memory, simultaneously. The water maze relies on aversive escape motivation, which generates a stress response that likely confounds experimental designs [20]. Indeed, this stress response has been shown to underlie sex differences in water maze training [19] and to differentially affect performance on a variety of other cognitive tasks [21–23]. Although the RAM requires mild food deprivation, which can impact some measures of anxiety in male but not female rats [24], there is no evidence that food deprivation by itself elicits sex differences in RAM performance or motivation. 

Additionally, the RAM allows for the examination of sex-differences in two memory systems, reference and working memory. Reference memory is associated with long-term memory of the task rules. In the case of the RAM, it refers to the stationary location of the baited arms, which remains constant across all trials. Working memory, in contrast, requires rats to take into account the arms they have or have not visited during each training trial [25,26]. In order to visit each arm only once and minimize errors, working memory must be maintained until all the food rewards are retrieved in a single trial and then reset for the next trial. 

Furthermore, we employed a protocol whereby the orientation of the rats upon release is randomized between trials but the experimenter remained in the same location when animals were released. This design has been shown to eliminate differences in spatial learning between sexes [4,27,28]. We combined this paradigm with a robust training protocol, involving 10 consecutive trials on the maze for six consecutive days to diminish acquisition differences. Robust training involving multiple trials has been shown to reverse short-term memory deficits and eliminate spatial memory deficits altogether with subsequent re-training in memory-impaired transgenic mice [29–31]. Additional evidence has demonstrated that overtraining or training to criteria minimizes memory deficits in older animals [32–34].

While the neurochemical underpinnings of learning and memory has a rich and thriving literature, much of this research has relied exclusively on male subjects. Recently, some reports have identified specific differences in neurochemistry between sexes, identifying activation of CREB as particularly important for male but not female memory [35,36]. Here we investigate the effects of two synaptic markers that are known to be important for the maintenance of long-term spatial memory, protein kinase M zeta (PKMζ) and the α-amino-3-hydroxy-5-methyl-4-isoxazolepropionic acid (AMPA) receptor subunit GluA2. Studies have shown that PKMζ is important for maintaining the late-phase of LTP [37,38], by increasing the AMPA receptor numbers, specifically those containing GluA2 subunits [39,40]. Interestingly, the PKMζ gene is known to have CREB binding sites [41]. In addition, PKMζ has also been shown to be important for maintaining long-term memory for spatial location [42–45], taste aversion [46,47], and fear conditioning [43]. Thus, the objective of the current study is to identify whether there are sex differences in remote long-term retention and characterize how they correspond to different expression patterns for PKMζ and GluA2.

## Materials and Methods

### Subjects

Adult male and female 8-week old Sprague-Dawley rats (Harlan; Indianapolis, IN) were used for the current experiment (n=6 per group, 60 rats total). Rats were pair-housed in plastic cages (48 x 27 x 16 cm) containing hardwood bedding. Animal quarters were maintained at constant temperature (22±1°C) and relative humidity (40-50%) with a 12h light/dark cycle (lights on at 8AM). Food (Harlan Teklad; Frederick, MD) and water were available *ad libitum* prior to behavioral training. All procedures were performed in accordance with the NIH Guide for the Care and Use of Laboratory Animals and approved by the Institutional Animal Care and Use Committee at Hunter College.

### Behavioral procedure

Rats were trained on the radial arm maze as previously described [43,48]. The maze consisted of a central platform (38cm diameter) and eight radiating arms (70 x 11 cm). Prior to training, rats were food-restricted to gradually reach 85% of their free-feeding weight and then shaped for 3 days. Shaping consisted of three trials per day, during which they were habituated to the maze and to the sweetened oatmeal mash, which served as a food reward. For each shaping trial, rats were released individually in the center of the maze and allowed 10 minutes to forage and collect food from all arms. After shaping, each rat was randomly assigned four arms to be baited for the remainder of the experiment. During training, the assigned arms were each baited with 0.1g of oatmeal placed at the end of each arm and the other remaining 4 arms were unbaited. The sequence of baited/unbaited arms remained constant throughout the experiment for each subject. To prevent the use of internal cues, the maze was rotated 90° daily while the spatial location of the baited arms with respect to the room cues remained constant. Additionally, between every trial, the maze was wiped down with 70% ethanol to control for odor cues. During training, rats were placed onto the center of the maze and confined with a black box (20 x 20 x 20cm) prior to the beginning of each trial. Once released, the rat remained on the maze until it collected food from all baited arms or until 3 minutes had elapsed. The sequence of arms entered and the latency to find all four food rewards was recorded. A percent correct score and a tally of total errors were calculated for every trial. Rats made two types of errors: reference memory errors (entries into unbaited arms) and working memory errors (repeated entries into previously explored arms). Training occurred over 3 days (30 total trials) or 6 days (60 total trials), followed by a retention test either one day or 30 days after the last training trial. During retention testing, subjects were given 3 additional trials on the maze, using the same parameters as the training trials. 

### Tissue preparation

Immediately after their last retention trial, subjects were rapidly decapitated, trunk blood was obtained and brains were removed for hippocampal dissections. Hippocampi were stored at -80°C until processed for fractionation. Fresh frozen hippocampi were homogenized in TEE buffer containing protease and phosphatase inhibitors and spun at low speed (3,000g for 5min at 4°C) to remove the nuclear pellet. Samples were then ultracentrifuged (100,000g for 30min at 4°C) to separate out the cytosolic fraction in the supernatant [49]. The remaining pellet was resuspended in homogenizing buffer containing 0.001% Triton X-100, incubated on ice for 1h and then spun in the ultracentrifuge (100,000g for 1h at 4°C). The pellet from this spin is the synaptic fraction [50]. Total protein concentrations for each fraction were determined using a BCA assay (Pierce; Rockford, IL) and standardized for Western blotting. 

### Western blotting

Samples were subjected to SDS-PAGE and transferred to nitrocellulose membranes. The membranes were incubated overnight at 4°C with primary antibodies selective for: PKMζ (1:1000, Santa Cruz Biotechnology; Santa Cruz, CA) and GluR2 (1:1000, EMD Millipore; Billerica, MA). After incubation with appropriate HRP-conjugated secondary antibodies, the reaction product was visualized using ECL substrate (Thermo Scientific; Pittsburgh, PA). GAPDH (1:2000, EMD Millipore; Billerica, MA) was used as a loading control. The membranes were scanned and band densities were measured using ImageJ (NIH; Bethesda, MD).

### Statistical analyses

Performance on the radial arm maze during the training period was analyzed using two-way, repeated measures ANOVAs. Learning curves for percent correct, reference memory errors and working memory errors were analyzed as trial blocks (5 trials each, 2 trial blocks/day). Performance during the retention test and Western blot data were analyzed using three-way ANOVAs for sex, training and retention period. Where there were significant main effects, we further compared specific groups using 1-way ANOVAs. Relationships between retention test performance and PKMζ or GluA2 expression were analyzed using Pearson’s correlations.

## Results

### Radial arm maze acquisition

**Figure 1:**
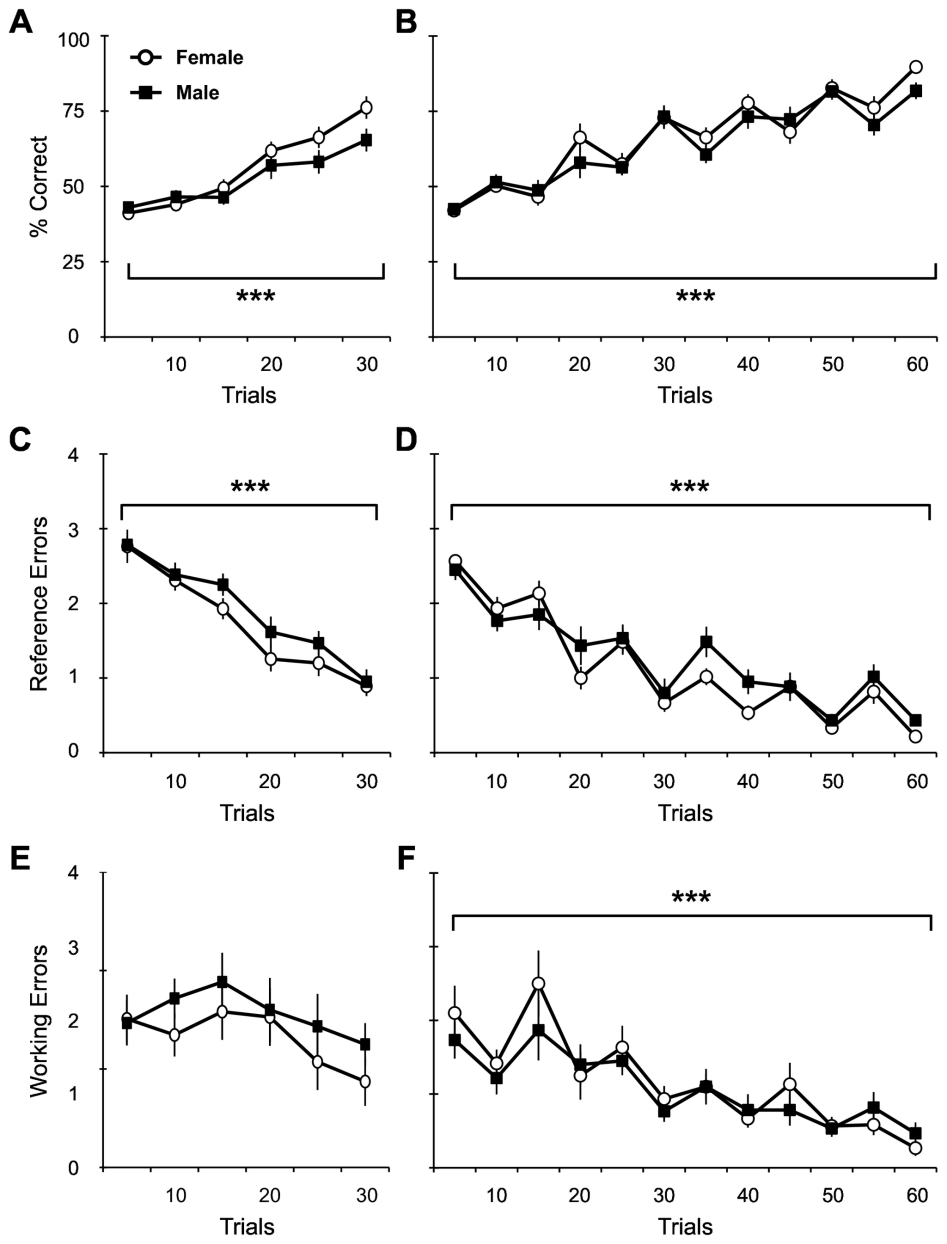
RAM training produced equivalent learning between sexes with either 30 or 60 training trials. (A, B) There was an overall significant improvement in percent correct over time and no significant differences with respect to sex in subjects given either 30 or 60 trials during training. However, the 60-trial cohorts achieve asymptotic performance during the last 2 days of training trials but the 30-trial cohorts do not. (C, D) The average number of reference memory errors per trial was significantly reduced over time with no sex effects in subjects given either 30 or 60 trials during training. (E, F) The average number of working memory errors per trial was significantly reduced over time only in subjects given 60 trials during training. There were no sex differences in either 30- or 60-trial cohorts. For all graphs, asterisk denotes significant effect over time (***p<0.001).

Male and female rats received 30 or 60 RAM training trials (n=12 per group). The percent correct score shown in Figure 1A-B reflects the number of correct arm entries (i.e. baited arm entrances) divided by the total number of arm entries within a single trial. Male and female rats trained for 30 trials (Figure 1A) demonstrated significant improvement over time (F_5,105_ = 26.20, p<0.0001), but no significant differences with respect to sex (F_1,105_ = 3.86, p = 0.063). Subjects trained for a total of 60 trials (Figure 1B) also demonstrated significant improvement over time (F_11,242_ = 42.49, p<0.0001), without any significant differences between sexes (F_1,242_ = 0.85, p = 0.366). While all cohorts of animals showed significant improvement over the entire training period, when analyzed for asymptotic performance during the last 2 days of training, only subjects trained for 60 trials demonstrated it, with no differences in percent correct scores over time (day 5 vs. day 6: F_1,22_ = 2.09, p = 0.163). This was not the case with the 30-trial subjects, which continued to show improvement in percent correct scores over time (day 2 vs. day 3: F_1,22_ = 26.32, p<0.0001).

Further analysis of errors made during training confirmed these effects (Figure 1C-F). Reference memory errors are the errors made when an arm is entered that is never baited. Male and female rats trained for 30 trials (Figure 1C) showed a significant decrease in reference errors over time (F_5,105_ = 37.89, p<0.0001). These errors were not significantly different between sexes (F_1,105_ = 3.04, p = 0.096). Subjects that received 60 trials during training (Figure 1D) also showed a significant decrease in reference memory errors over time (F_11,242_ = 47.21, p<0.0001) with no difference between sexes (F_1,242_ = 1.26, p = 0.274). When analyzed for asymptotic performance during the last 2 days of training, subjects trained for 30 trials demonstrated continued improvement in reference errors over time (day 2 vs. day 3: F_1,22_ = 46.32, p<0.0001), while 60-trial subjects reached asymptotic performance, with no further improvement over time (day 5 vs. day 6: F_1,22_ = 0.01, p = 0.913).

Interestingly, working memory errors did not significantly change over time in male and female rats trained for 30 trials (Figure 1E; F_5,105_ = 1.67, p = 0.148), nor were there any significant differences between sexes (F_1,105_ = 2.05, p = 0.167). This error type is committed when a previously entered arm is re-entered within a trial. Rats trained for 60 trials did show a significant decrease in working memory errors over time (Figure 1F; F_11,242_ = 5.80, p<0.0001). There were no differences in working memory errors between sexes for 60-trial subjects either (F_1,242_ = 0.37, p = 0.551). Lastly, subjects trained for 30 trials did show improvement in working errors over time (day 2 vs. day 3: F_1,22_ = 8.40, p<0.01), while 60-trial subjects reached asymptotic performance, with no further improvement over time (day 5 vs. day 6: F_1,22_ = 2.79, p = 0.109).

### Radial arm maze retention

**Figure 2:**
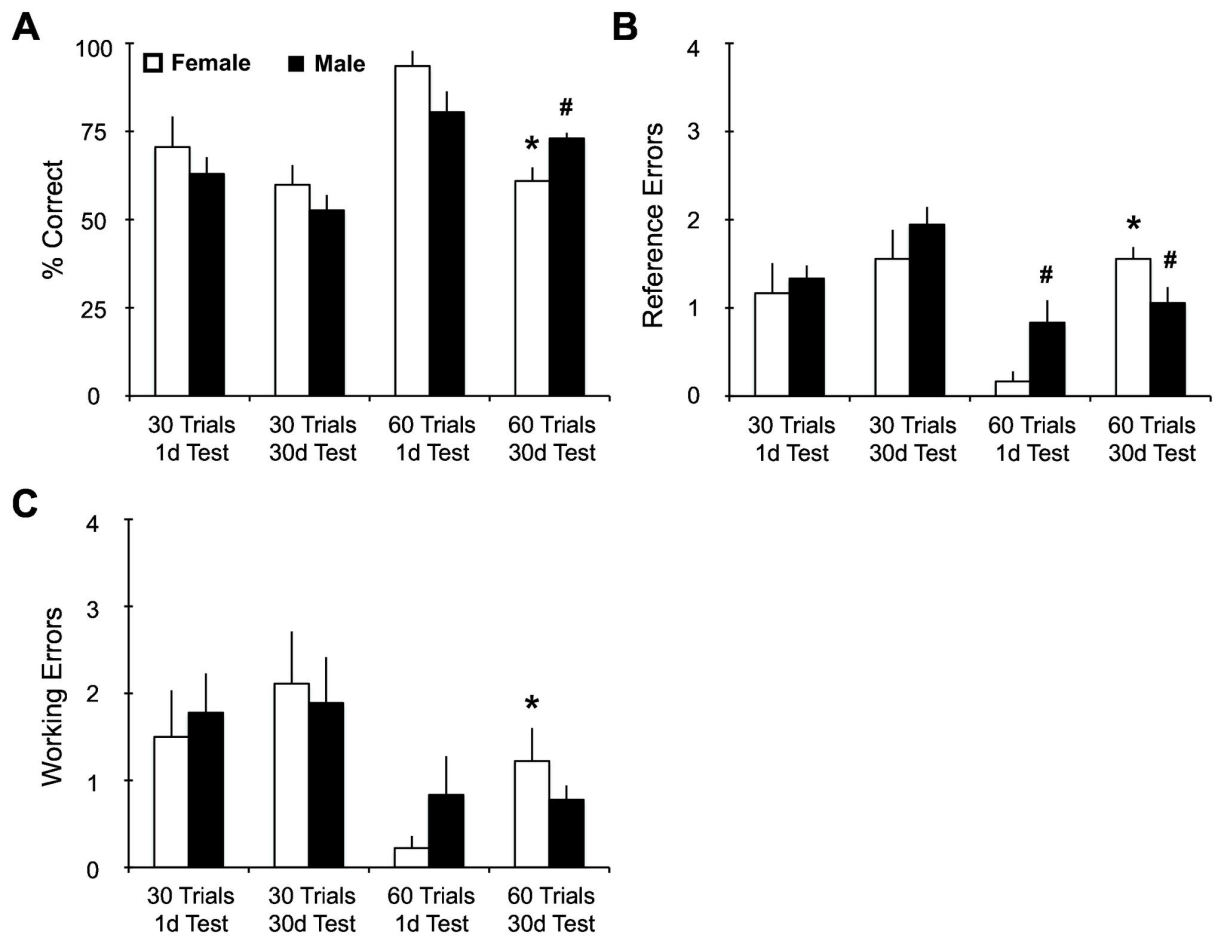
Memory retention test scores1d and 30d post training. (A) Overall performance on the retention test showed significant training and retention period effects. The 60-trial cohorts showed significantly better retention compared to 30-trial cohorts. In the post-hoc analysis, there were no differences between sexes in the 60 trial / 1d test condition. Females in the 60 trial / 30 d test group showed significantly lower retention scores compared to males of the same condition and compared to their female 1d counterparts. (B) Reference memory errors showed significant training and retention period effects. The post-hoc analysis showed that females trained for 60 trials and tested 30d later make significantly more reference errors compared to males of the same condition and compared to their female 1d counterparts. (C) Working memory errors revealed significant training effects, with 60-trial cohorts making significantly fewer errors compared to 30-trial cohorts. In the post-hoc analysis, females trained for 60 trials and tested 30d later showed a significant increase in working errors compared to 60 trials / 1d test females. For all graphs, pound (#) denotes sex significance compared to female counterparts in the same training/testing condition. Asterisk (*) denotes significance compared to same-sex counterparts in the 60 trial / 1d test condition.

All rats had a retention test either 1d or 30d after completing training. Retention test scores (Figure 2A), calculated from the average of 3 test trials, reflect overall significant effects with training (F_1,40_ = 16.11, p<0.0001) and retention period (F_1,40_ = 18.32, p<0.0001). Male and female rats trained for 60 trials and tested 1d later had the highest percent correct scores (93.5±4.3 female; 80.4±5.9 male). Female rats trained for 60 trials and tested 30d later had a significant reduction in memory retention (58.5±3.9) compared to females in the 60 trials / 1d test group (F_1,10_ = 36.67, p<0.0001) and compared to males in the 60 trials / 30d test group (F_1,10_ = 11.93, p<0.01). Male rats trained for 60 trials and tested 30d later did not show significantly different percent correct scores (73.0±1.6) compared to males in the 60 trials / 1d test group. These retention data suggest that females from the 60 trial / 30d test condition have weak memory retention score. Both male and female rats trained for 30 trials and tested 30d later did not show any significant reductions in performance (58.7±6.7 female; 52.6±4.4 male) compared to subjects trained for 30 trials and tested 1d later (70.6±8.7 female; 62.9±4.8 male). Without having reached asymptotic performance in the 30-trial cohorts, individual learning differences within conditions are often larger, undermining a significant group effect. This interpretation is consistent with our overall training effect, identifying the 60-trial subjects as performing significantly better than our 30-trial subjects. 

There was also overall significant effects in reference memory errors (Figure 2B) with training (F_1,40_ = 11.87, p<0.001) and retention period (F_1,40_ = 18.55, p<0.0001), as well as a sex/training/retention period interaction (F_1,40_ = 5.82, p<0.05). Males trained for 60 trials and tested 1d later showed significant increases in reference errors compared to females in the same treatment group (F_1,10_ = 5.71, p<0.05). Females trained for 60 trials and then tested 30d later showed significant increases in reference errors compared to males in the same condition (F_1,10_ = 8.78, p<0.05) and females in the 60 trials / 1d test group (F_1,10_ =78.40, p<0.0001). These statistical differences were specific to the 60-trial cohorts, where asymptotic performance was observed during training (Figure 1). When acquisition curves do not reflect asymptotic performance (30-trial cohorts), there were no differences in reference errors between males and females tested either 1d or 30d later. This is consistent with their overall performance during test (Figure 2A). Analysis of working memory errors (Figure 2C) during retention testing revealed an overall training effect (F_1,40_ = 11.42, p = 0.01). Females trained for 60 trials and tested 30d later made more working errors compared to their counterparts in 60 trials / 1d test group (F_1,10_ = 6.81, p<0.05), an effect likely the result of the very high retention score and low working memory errors of the female 60 trial / 1d test condition. There were no overall sex differences in working errors. 

### PKMζ and GluA2 (AMPA receptor subunit) group analysis

**Figure 3:**
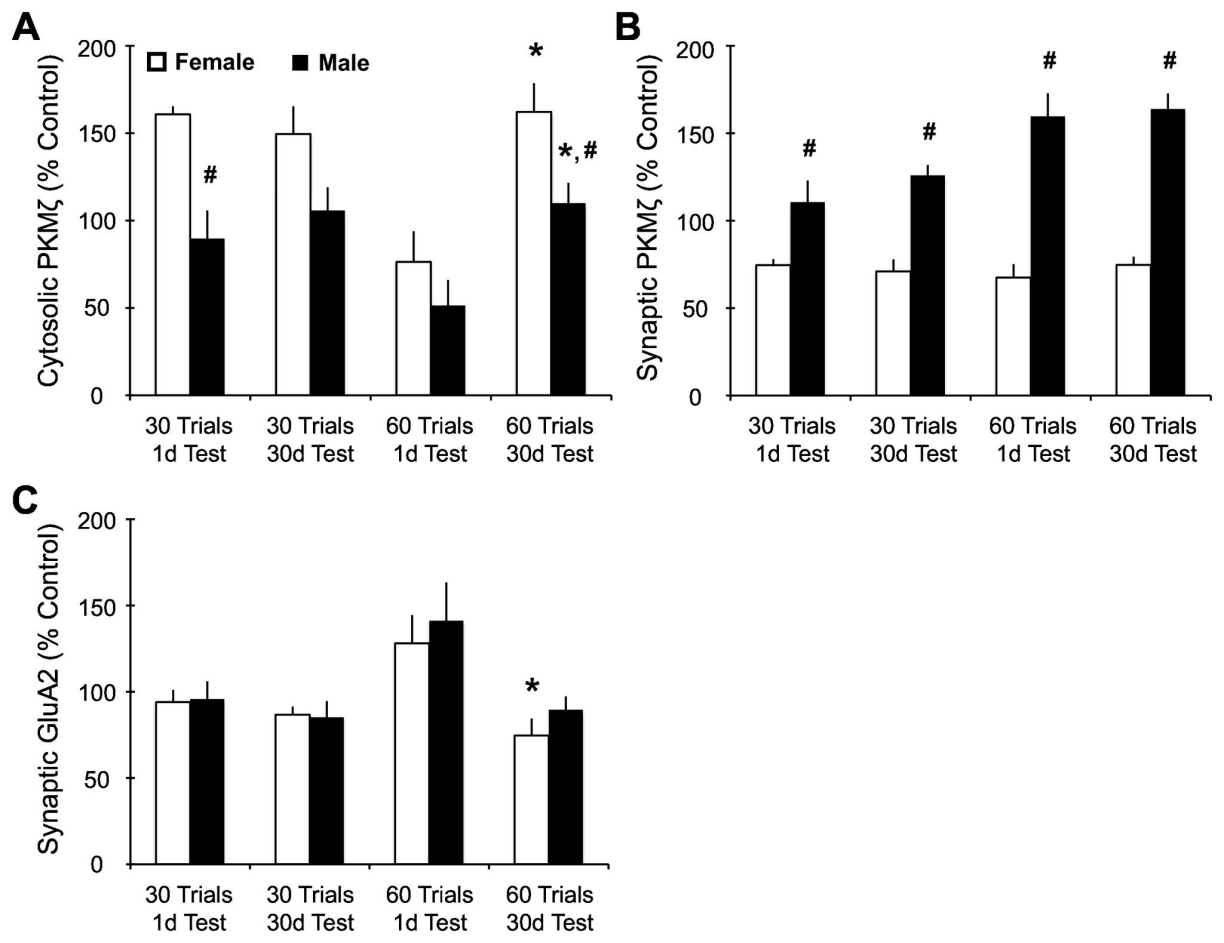
RAM training induces differential PKMζ and GluA2 expression between sexes in the hippocampus. (A) Cytosolic PKMζ levels showed significant sex, training and retention period effects. In the post-hoc analysis, both males and females trained for 60 trials and tested 30d later showed a significant increase in cytosolic PKMζ expression compared to 60 trials / 1d test subjects. Females from both the 60 trials / 30d and 30 trial / 1 d condition showed significantly higher levels of PKMζ compared to males of the same conditions. (B) Synaptic PKMζ levels showed significant sex and training effects. Across all conditions males show significantly higher levels of synaptic PKMζ compared to females. (C) Synaptic GluA2 levels showed significant training and retention period effects. In addition, females trained for 60 trials and tested 30d later showed a significant decrease in GluA2 expression compared to 60 trials / 1d test females. For all graphs, pound (#) denotes significant sex difference compared to females in the same training/testing condition. Asterisk (*) denotes significance compared to same-sex counterparts in the 60 trial / 1d test condition.

Immediately following retention testing, the hippocampus from each rat was removed, fractionated to obtain the cytosolic and synaptic regions of the cell and probed for expression of PKMζ and GluA2 by Western blotting. The expression of these markers in both the cytosolic and synaptic fractions are shown as percent of untrained male and female controls. Cytosolic PKMζ (Figure 3A) demonstrated an overall significant effect on retention (F_1,37_ = 13.58, p<0.0001), training (F_1,37_ = 6.85, p<0.001) and sex (F_1,37_ = 22.51, p<0.0001), and a training by retention interaction (F_1,37_ = 11.88, p<0.001). 

Both males and females cohorts from the 60 trials / 1d test had the lowest levels of cytosolic PKMζ and the highest levels of memory retention. The 60 trial / 30d test showed a significant increase in PKMζ compared to their counterparts tested 1d after training (female: F_1,10_ = 12.72, p<0.01; male: F_1,10_ = 9.76, p<0.05). Additionally, the females given 60 trial / 30d test showed significantly higher levels of cytosolic PKMζ compared to males in the same condition (F_1,10_ = 6.68, p<0.05). Females in the 30 trial / 1d test cohort showed significantly higher levels of cytosolic PKMζ compared to their male counterparts (F_1,9_ = 21.29, p<0.001). This sex effect is not observed in the 30 trial / 30d test group, suggesting differential expression of cytosolic PKMζ during memory formation but not retention after non-asymptotic performance. However, more direct studies will be needed to delineate this result. 

The level of PKMζ expressed within the synaptic fraction (Figure 3B) revealed overall effects for sex (F_1,37_ = 142.40, p<0.0001) and training (F_1,37_ = 13.44, p<0.001), with a sex by training interaction (F_1,37_ = 15.68, p<0.0001). Males had higher synaptic PKMζ expression compared to females for all groups: 30 trials / 1d test (F_1,9_ = 9.31, p<0.05), 30 trials / 30d test (F_1,9_ = 35.80, p<0.0001), 60 trials / 1d test (F_1,9_ = 39.79, p<0.0001) and 60 trials / 30d test (F_1,9_ = 86.86, p<0.0001). These data identify the expression level of synaptic PKMζ as a marker that is differentially expressed between sexes, irrespective of whether the memory retention was developed after asymptotic performance.

The expression of the AMPA receptor subunit GluA2 within the synaptic fraction (Figure 3C) also revealed overall significant effects for training (F_1,36_ = 4.71, p<0.05) and retention (F_1,36_ = 13.83, p<0.001), with a training by retention interaction (F_1,36_ = 6.95, p<0.05). Surprisingly, there were no differences in synaptic GluA2 expression between males and females in any of the groups. Females trained for 60 trials and tested 30d later showed a significant decrease in GluA2 compared to females trained for 60 trials and tested 1d later (F_1,9_ = 8.43, p<0.05). Admittedly, this decrease would have stronger implications with additional statistical differences between sex in the 60 trial / 30d test condition. However, it is interesting that the females in the 60 trial / 30d condition have a reduction in GluA2 and reduced memory retention. Together these data suggest that the females trained and tested 1d later may be using different mechanisms to upregulate GluA2, in lieu of increasing PKMζ within the synaptic fraction. 

### Synaptic PKMζ and GluA2 correlation analysis

**Figure 4:**
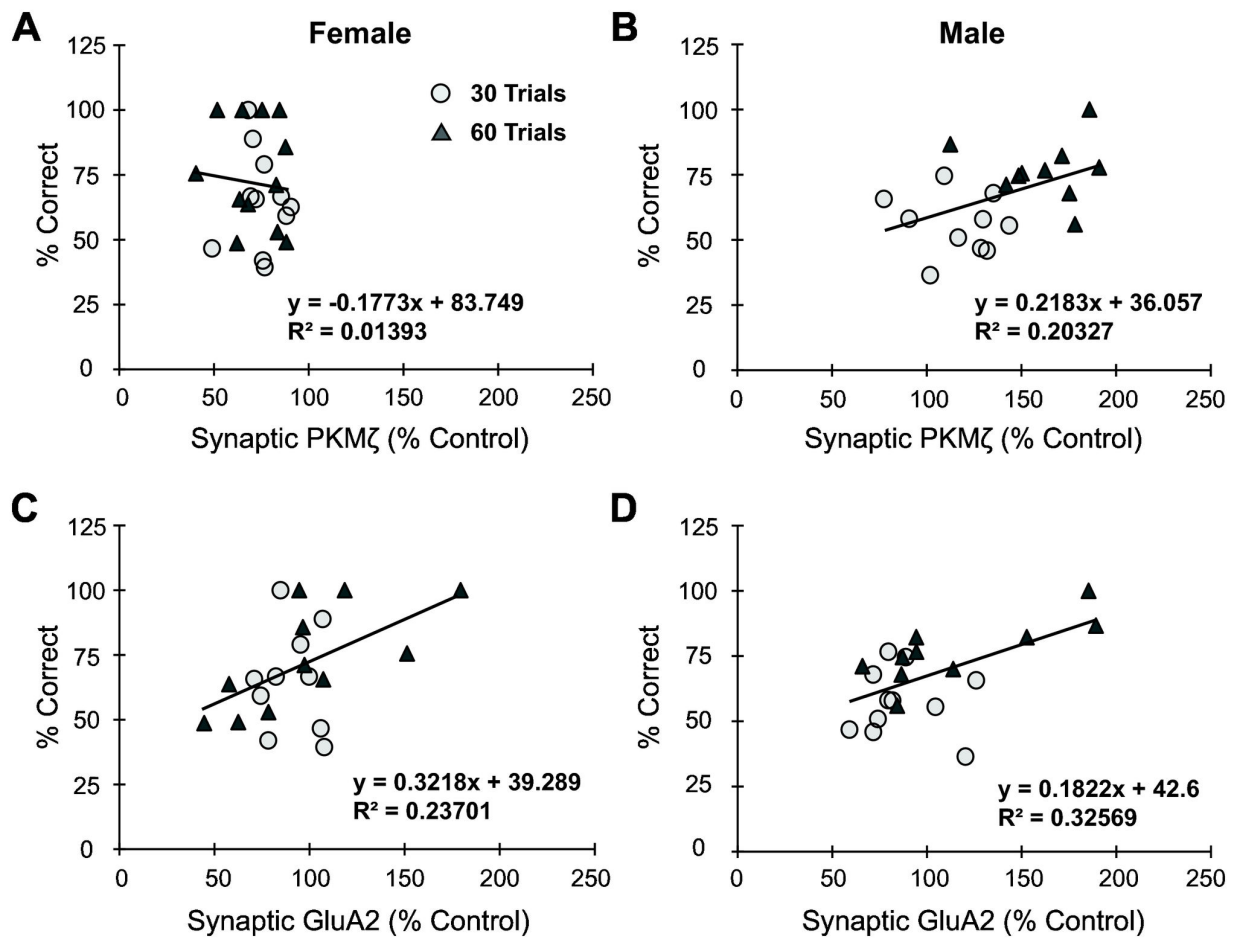
Synaptic PKMζ and GluA2 expression differentially correlates with memory retention between sexes. (A, B) Males, but not females, showed a significant correlation between retention test performance (percent correct scores) and synaptic PKMζ levels in the hippocampus. (C, D) Both males and females showed a significant correlation between retention tests scores and synaptic GluA2 levels in the hippocampus.

After combining all training and testing conditions together, neither males nor females showed a significant correlation between retention test scores (percent correct) and cytosolic PKMζ expression (data not shown). In contrast, males, but not females, showed a significant correlation (Figure 4A-B) between retention test scores and synaptic PKMζ expression (R = 0.451, p<0.05). In addition, both males and females showed a significant correlation (Figure 4C-D) between retention score and synaptic GluA2 (female R = 0.488, p<0.05; male R = 0.571, p<0.01). These correlations, involving all the subjects from both 30- and 60-trial cohorts, strengthen our understanding of synaptic markers and their participation in memory, particularly in the 30-trial cohorts where group effects were not significant.

## Discussion

The objective of the current report was to characterize sex differences in spatial remote (30d) long-term memory and their underlying neurochemistry involving PKMζ and GluA2 expression. We utilized a robust training protocol (60 training trials) on the radial arm maze to attenuate the sex differences in acquisition of spatial information, so that we could examine sex difference in memory retrieval, specifically in retention testing 30d after training. Females trained for 60 trials performed significantly worse when tested 30d after training compared to females tested 1d after training, by making more references and working memory errors. These females (60 trial / 30d test) also performed significantly worse than their male counterparts for both percent correct and reference errors (Figure 2A-B). Males given 60 training trials did not perform differently when tested 1d or 30d after training. These data show that, with asymptotic performance on the RAM, females outperform males in making significantly fewer reference errors at the 1d test. However, at 30d test, females showed a significant deficit compared to their male counterparts. This suggests that males do no retain spatial information as well as females do 24h post-training but maintain this spatial information for longer. 

Unsurprisingly, both sexes demonstrated a training effect at test, with 30-trial subjects performing significantly worse compared to 60-trial subjects and making more reference and working memory errors. Similarly, both sexes demonstrated an overall retention period effect, performing significantly worse when tested 30d after training compared to 1d. The differences in memory retention observed between 30 and 60 training trials could be interpreted by whether asymptotic performance had been achieved. Thus, it is expected that in cohorts where asymptotic performance was not achieved (i.e. 30 training trials), there will be greater variability between subjects as rates of learning vary between animals. These differences are often reduced as the cohort achieves asymptotic performance. Therefore, we expected that there would be fewer significant differences between memory retention tests at 1d compared to 30d following 30 training trials. 

Analysis of PKMζ expression showed overall sex effects, with females showing increased cytosolic PKMζ compared to males. Both sexes in the 60 trial / 1d test cohorts performed the well at test but had the lowest levels of cytosolic PKMζ. These results suggest that spatial training resulting in asymptotic performance may decrease cytosolic PKMζ for both sexes. It appears that as this robust memory is weakened over time, the levels of cytosolic PKMζ associated with retention testing increase for both sexes. Behaviorally, males given 60 training trials did not show significant differences between the 1d and 30d test but did show significant changes in the cytosolic PKMζ levels. This discrepancy could reflect the sensitivity in the molecular memory that is not registered in the behavioral retention test data. It is interesting to speculate that the pattern of cytosolic expression between sexes in the 60 trial / 30d test condition is similar to that of the 30 trial / 1d test. This suggests that the molecular memory at of the 60 trial / 30d test condition is similar to that of the 30 trial / 1d test. Future experiments will examine the levels of PKMζ with and without retention testing to delineate these effects. 

Synaptic PKMζ expression significantly increased in all males compared to females. Interestingly, only male cohorts appeared to positively correlate synaptic PKMζ expression with memory retention. Cohorts where asymptotic performance had been reached (60 training trials) reflected the highest levels of synaptic PKMζ compared to non-asymptotic performance cohorts (30 training trials). 

We propose that the expression of synaptic PKMζ observed in male rats acts as a molecular mechanism for remote (30d) long-term spatial memory. This relationship between long-term spatial memory and PKMζ is further strengthened by the significant positive correlation between synaptic PKMζ and improved retention test scores in males. Additionally, there is a significant positive correlation with synaptic GluA2 expression and memory retention for both sexes, suggesting that females may rely on other mechanisms to increase synaptic GluA2, without increasing synaptic PKMζ.

### Sex differences in spatial acquisition and retention

Previous work examining sex differences in learning and memory has shown a male advantage in both rodents and humans on spatial maze tasks [1,2,51–53]. While some have argued against the validity of ascribing an evolutionary cause to explain such a distinction [2], the main effect still exists between sexes. Several reports have posited that the sex differences observed in spatial acquisition and retention are driven by distinct strategies preferred by each sex. Males utilize a geometry-based strategy, which relies on the interpretation of distal cues, while females preferentially utilize a landmark-based strategy, which focuses on nearby spatial markers [28,54–59]. In contrast, we utilized a robust training protocol to ensure equivalent performance during the acquisition phase of the task, in order to examine sex differences in retention, both the 1d and 30d post-training. It has been previously noted that consecutive training days involving massed trials can eliminate and/or mask sex differences that can be observed with fewer training trials [60]. In our studies, females given 60 training trials outperform males on the 1d retention test by making fewer reference errors and this difference is reversed in the remote long-term memory retention test. The differences observed in memory retention here may in part be driven by distinct acquisition strategies between sexes. This would suggest that the landmark strategy preferentially utilized by females might be less effective for remote (30d) long-term memory storage and retrieval, though this hypothesis remains to be tested.

### Protein kinase mobilization during learning and LTP

A significant aspect of this paper is determining the relationship between memory and the distinct cellular milieu where PKMζ is differentially expressed between sexes. We observed that the male and female cohorts differentially upregulated PKMζ expression, with both males and females showing increased cytosolic PKMζ but only males showing increased synaptic PKMζ for the 30d retention test which is also significantly higher than females in the same condition. Across all training groups, neither male nor female cohorts showed a significant correlation between retention test scores and cytosolic PKMζ expression. Conversely, only males showed a significant positive correlation between retention test scores and synaptic PKMζ. These data highlight the importance of protein kinase expression in the context of learning. Previous studies have shown that rodents with higher hippocampal levels of conventional PKCs within the particular fraction (associated with the post-synaptic density) after training have better spatial memory [50,61]. Conversely, low hippocampal levels of conventional PKCs are associated with poor spatial memory [62]. The dissociation between male cohorts (after 60 training trials) showing better remote memory compared to females in the same condition is consistent with how synaptic PKMζ is believed to function in late-phase LTP. As late phase LTP develops, higher concentrations of the myristoylated-ζ inhibitory peptide (ZIP) are required to reverse the potentiated response five hours after induction, suggesting that PKMζ is integrated with the synaptic membrane [38]. In contrast, increases in cytosolic PKMζ can occur within 15min to 3h after LTP [63]. These studies reflect how the mobilization of PKMζ from the cytosol to the synapse may occur over time as a mechanism involved in synaptic strengthening by integrating with protein substrates and/or cytoskeletal elements as seen with other kinases [64,65].

### PKMζ and GluA2 in synaptic plasticity and memory

Our results also showed that the AMPA receptor subunit GluA2 was equally expressed between sexes and significantly correlated with retention test scores. Previous work in male rats has shown that GluA2 stabilization within the membrane is a necessary function during memory consolidation, a process that is driven by increasing basal levels of PKMζ, but not increasing GluA2 levels per se [44]. This result is complimentary with electrophysiology studies showing that insertion of GluA2 receptors into the synapse is a necessary downstream consequence of PKMζ overexpression increasing EPSC [39,40]. In our study, females given 60 training trials demonstrated significantly decreased synaptic GluA2 levels 30d after training, when memory retention was significantly lower compared to males of the same condition. Females did not show a significant increase in synaptic PKMζ expression, nor was PKMζ expression associated with better memory performance. However, both sexes showed a decrease in cytosolic PKMζ levels at the 1d test following 60 training trials, suggesting that males and females differentially use PKMζ, depending on whether or not it is localized in the synapse. Thus, the increase in GluA2 that we find in our female cohorts may reflect a sex difference in memory processing and/or maintenance mechanisms. Interestingly, treatment of gonadectomized rats with estradiol results in a two-fold higher increase in hypothalamic GluA2/3 expression in females compared to males [66]. Recent studies have also highlighted other examples of sex-specific molecular activities for memory function. In transgenic mice expressing low-levels of p25, female but not male p25 mutants have improved spatial memory formation and enhanced LTP at hippocampal CA1 synapses [67]. Similarly, identical spatial training up-regulates hippocampal CREB phosphorylation and induces an up-regulation of hippocampal GAA1 mRNA involved in GPI anchoring of protein expression only in male WT mice [36]. These studies suggest that RAM memory may be driven by several molecular mechanisms that are activated differentially in male and female rats. 

## Conclusion

Our results indicate that a robust training protocol can be used to generate equivalent learning between male and female rats in the RAM paradigm. However, in spite of equivalent performance during acquisition, we found sex differences in the 1d test where females make significantly less reference errors compared to males and the reverse for the remote (30d) long-term memory. The male advantage in remote long-term memory was associated with a significant increase in synaptic PKMζ expression, as well a positive correlation between retention test performance and both synaptic PKMζ and GluA2 expression. In contrast, females had lower 30d retention scores compared to males and only a positive correlation between retention test performance and synaptic GluA2 expression. The present data suggest that altered utilization of PKMζ and GluA2 in a sexually dimorphic manner may readily influence retrieval for remote long-term memory. 
